# Experimental procedure for the characterization of radiation damage in macromolecular crystals

**DOI:** 10.1107/S0909049511002251

**Published:** 2011-03-10

**Authors:** Ricardo M. F. Leal, Gleb P. Bourenkov, Olof Svensson, Darren Spruce, Matias Guijarro, Alexander N. Popov

**Affiliations:** a ESRF, Rue Jules Horowitz, BP 220, Grenoble, France; b EMBL Hamburg Outstation, c/o DESY, Notkestrasse 85b, 22607 Hamburg, Germany

**Keywords:** *BEST*, *EDNA*, radiation damage

## Abstract

A reliable and reproducible method to automatically characterize the radiation sensitivity of macromolecular crystals at the ESRF beamlines has been developed. This new approach uses the slope of the linear dependence of the overall isotropic *B*-factor with absorbed dose as the damage metric. The method has been implemented through an automated procedure using the *EDNA* on-line data analysis framework and the *MxCuBE* data collection control interface. The outcome of the procedure can be directly used to design an optimal data collection strategy. The results of tests carried out on a number of model and real-life crystal systems are presented.

## Abstract 2

A novel automatic procedure to determine the sensitivity of macromolecular crystals to radiation damage is presented. The information extracted from this procedure can be directly used for optimal planning of data collection or/and beamline calibration.

## 1. Introduction

Radiation damage incurred during data collection in macromolecular crystallography (MX) limits the information that can be obtained from a single crystal. It occurs at any temperature and leads to a resolution-dependent reduction in diffraction intensity, changes in the unit-cell parameters and crystal mosaicity as well as slight rotations and translations of macromolecules in the lattice. It also induces specific chemical changes (*e.g.* disulphide bond breaks, decarboxylation of acidic residues, changes in the oxidation state of metal ions) which may prevent the structure solution or mislead biological interpretations. Comprehensive reviews of these topics are given by Garman & Owen (2006 ▶), Ravelli & Garman (2006 ▶) and Garman (2010 ▶).

Consideration of radiation damage effects is critical for optimal data collection planning. In the last decade significant progress has been made in the knowledge and understanding of the radiation damage phenomenon. Most of its manifestations are proportional to the absorbed dose and can be well predicted if the absorbed dose is known. Routine measurements of the X-ray beam flux and beam sizes are therefore of great importance. The overall isotropic *B*-factor has been found to be a robust measure of global radiation damage at 100 K. It shows a linear dependence with the absorbed dose and can be written as 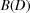 = 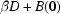, where 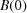 is the *B*-factor value with zero dose, 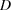 is the absorbed dose and 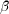 is a constant scale factor, representing the *B*-factor decay rate (Kmetko *et al.*, 2006 ▶; Bourenkov & Popov, 2006 ▶; Borek *et al.*, 2007 ▶, 2010 ▶).

In general, the same rate of decay (∼1 Å^2^ MGy^−1^) is observed for all protein crystals as seen in several independent investigations (Kmetko *et al.*, 2006 ▶; Bourenkov & Popov, 2010 ▶; Owen *et al.*, 2006 ▶; Holton, 2009 ▶). However, there is still a common opinion that some samples are more sensitive or more resistant to radiation than others (*e.g.* Pechkova *et al.*, 2009 ▶). In practice, apparent deviations in radiation sensitivity often arise not from a specific feature of the crystal structure but from a mismatched beam size, flux mis-calibration or other technical problems. When the sample sensitivity or beam calibration are uncertain, a reliable standardized procedure to calibrate a linear damage model is necessary through a preliminary experiment, sacrificing a whole or part of a sample. We have therefore established a new automatic procedure to determine the crystal sensitivity to radiation damage involving the measurement of the degree of damage in a sample or in part of it.

For the sake of reliability and transferability, we opted to implement this new development in the context of the *EDNA* on-line data analysis platform (Incardona *et al.*, 2009 ▶). *EDNA* is a framework for developing plug-in-based applications especially designed for X-ray experiments. It is now reaching a mature stage with a set of well defined plug-ins to invoke common data processing tasks (*e.g.* data indexing and integration, and data collection strategy) and a set of test cases to ensure software reliability. *EDNA* has been recently integrated in the ESRF beamline control interface, the *MxCube* software (Gabadinho *et al.*, 2011 ▶), allowing for ‘one click’ sample characterization. This feature fully characterizes the crystal sample and generates a data collection strategy that accounts for radiation damage. Here we present the development and testing of an automated procedure for the determination of the radiation damage rate, providing calibration and verification of a linear *B*-factor decay model. The information extracted from this procedure can be directly used for optimal planning of data collection while accounting for radiation damage in data collection planning software, such as *BEST* (Bourenkov & Popov, 2010 ▶). Using test crystals with well known radiation sensitivity, the procedure can also be used at the beamlines to verify and calibrate flux and beam size.

## 2. Methods

### 2.1. Data collection protocol

The procedure for characterization of radiation damage aims to describe the variation in scattering power (diffracted intensity and isotropic *B*-factors) with exposure time in a reliable and reproducible way. It was developed to suit crystals having a broad range of diffraction quality.

The data collection protocol is generated automatically on the basis of data obtained from the initial sample characterization step (see §2.2), and assumes that both the absorbed dose rate and crystal sensitivity (β ≃ 1 Å^2^ MGy^−1^) are approximately known. The protocol does, however, allow for deviations in sensitivity (or, equivalently, in dose) by up to a factor of ∼3. The experimental part consists of 11 successive collections of narrow wedges of data (the *collecting* cycle), interleaved by long X-ray exposures to ‘burn’ the crystal (the *burning* cycle). The protocol defines a complete set of required parameters: exposure time, attenuator transmission, total rotation range, rotation range per frame and resolution limit (*d*_min_) for data collections, and exposure time for irradiation.

As previously discussed by Kmetko *et al.* (2006 ▶), the variations in illuminated crystal volume during data collection may corrupt the analysis of radiation damage, as undamaged (or little damaged) parts of the crystal move in and out of the beam. In order to minimize the influence of such a non-homogeneous irradiation, the total crystal rotation range should be kept relatively narrow (Sliz *et al.*, 2003 ▶; Schulze-Briese *et al.*, 2005 ▶). On the other hand, the number of measured reflections has to be sufficiently large to achieve reliable *B*-factor estimations. We find that a total rotation range between 3° and 5° provides an adequate compromise in most practical cases. For the burning cycle the crystal is rotated within the same total rotation range as during data collection.

The rotation range is centred on a rotation angle used in the initial characterization step. Selecting a particular orientation is often required owing to the specific crystal habit, crystal visibility in a mounting loop/mesh or other practical details of the experiment, *e.g.* for very small crystals slight mis-centering on the rotation axis may result in detrimental variations in the dose rate with orientation. Thus our procedure assumes that the initial crystal orientation is carefully selected by the user on the basis of microscope images. This initial orientation is preserved throughout the whole procedure.

The absorbed dose in each of the collecting cycles is chosen never to exceed 0.1 MGy, so that the radiation damage induced at this step is relatively small. This consideration, combined with a standard *BEST* calculation as described by Popov & Bourenkov (2003 ▶), gives rise to a consistent choice of the resolution limit, exposure time and rotation width per frame. These deliver data with a predefined signal-to-noise ratio in the last resolution shell and without spatial overlap of reflections. For the first data set collected (before the first burning irradiation) we specify the signal-to-noise 〈*J*〉/〈σ_*J*_〉 = 5 in the last resolution shell. Also, we keep *d*_min_ = 2.0 Å, even if the crystal quality permits the collection of higher-resolution data within the given dose limit. This results in higher 〈*J*〉/〈σ_*J*_〉 in the last resolution shell. The attenuator transmission settings are adjusted according to the rotation speed and exposure time limitations defined by the diffractometer.

Following the model assumptions (*i.e.* approximately known dose rate and β ≃ 1 Å^2^ MGy^−1^), the dose for the burning cycles is selected in such a way that significant changes in *B*-factors are induced and, simultaneously, the intensity measurements remain statistically significant up to the last cycle of data collection. The total absorbed dose is chosen to reduce the intensity in the last resolution shell by approximately a factor of 3. For a strongly diffracting crystal, this value is approximately 10 MGy, *i.e.* one-third of the ‘Garman limit’ (Owen *et al.*, 2006 ▶). Such a choice for the burning dose ensures that sufficiently informative data are available even when the dose rate or β value are significantly under, or over, estimated. Note that higher doses are used for crystals diffracting to lower resolutions. This is consistent with a strongly resolution-dependent intensity decay model. Typically, for weakly diffracting crystals, the resulting 〈*J*〉/〈σ_*J*_〉 in the last resolution shell for the last data set is approximately 2, ensuring both the data quality and the capability to integrate the diffraction images without strong bias.

### 2.2. Implementation

Overall, the algorithm involves the following sequence of steps, each implemented using core software package(s) indicated in parentheses:

(*a*) collect reference images (*MxCuBE*);

(*b*) process (index and integrate) reference images (*MOSLFM*; Leslie, 1992 ▶);

(*c*) initial dose rate estimation (*RADDOSE*; Murray *et al.*, 2004 ▶; Paithankar *et al.*, 2009 ▶);

(*d*) generate a protocol for data collection/irradiation sequence (*BEST*);

(*e*) implement collection/irradiation sequence (*MxCuBE*);

(*f*) integrate the data [*XDS* (Kabsch, 2010 ▶) or *MOSFLM*];

(*g*) determine the overall scale and *B*-factors (*BEST*);

(*h*) generate plots of *B*-factors and relative scale *versus* dose; estimation of β using linear fitting (*Matplotlib*; http://matplotlib.sourceforge.net/).

The data exchange between the individual steps of the procedure and the execution of data processing sequences are implemented within the *EDNA* platform. As a temporary exception, data-processing steps (*f*)–(*h*) were achieved through a stand-alone Python script during the tests described in §3. Its integration into *EDNA* is currently being completed.

The first steps (*a*)–(*d*) of the procedure are integrated into an automated sample characterization functionality available in *EDNA* (Incardona *et al.*, 2009 ▶) which is invoked through the *MxCuBE* interface. The *EDNA* MXv1 characterization encapsulates plug-ins responsible for indexing and integration, dose rate estimation and data collection strategy planning. The radiation damage characterization is available to the user as one of the strategy options. The user defines the conditions [rotation range(s), number or frames, detector distance, exposure time] of the reference images, or chooses one of the standard protocols (such as two images 90° apart). Optional inputs are the crystal space group to be enforced on the indexing solution and the exact chemical composition of the crystal, which can be taken into account in the dose rate calculations.

By default, the chemical composition of an ‘average protein crystal’ (47% solvent content, 0.05 sulfurs per amino acid residue, 300 m*M* sulfur in the buffer solution) is passed to *RADDOSE* along with the beam flux and beam dimensions. Unless strong deviation from the average in the absorption properties is expected (*e.g.* owing to the presence of heavy atoms), the default composition is fully suitable for the purpose of radiation damage characterization on a relative scale, as required for data collection strategy optimization. For comparative studies, *e.g.* on radiation sensitivity of different samples or under different experimental conditions, or for an approximate flux density calibration, the exact chemical composition must be used.

The *EDNA* characterization routine results in a data collection protocol, as described in §2.1, which has the structure of a standard *EDNA* multi-wedge data collection object, modified to incorporate the irradiation steps. The generated protocol is automatically loaded back into the *MxCuBE* data collection queue, presented to the user for approval (with an editing option) and then executed.

Once the diffraction images are acquired, they are integrated using either *MOSFLM* or *XDS*. The integration process can optionally use the indexing solution inherited from the characterization step, ensuring consistency in processing even for difficult indexing cases. Integrated data are then again passed to *BEST* which determines the overall scales and isotropic *B*-factors by maximum-likelihood scaling to a generalized external reference (Popov & Bourenkov, 2003 ▶). Finally, both the relative scales and *B*-factors are plotted against the nominal dose using the Python library *Matplotlib*. Linear fitting of the *B*-factors *versus* dose is used to calculate the decay rate (or sensitivity coefficient) β. Displaying the plots provides a fairly intuitive overall indicator of the success or failure of the procedure.

## 3. Testing

Six crystal systems were selected for testing the method: thermolysin from *Bacillus thermoproteolyticus* (Mueller-Dieckmann *et al.*, 2007 ▶); bovine pancreatic trypsin (Bartunik *et al.*, 1989 ▶); a ten base pair oligonucleotide d(AGGGGCCCCT)_2_ A-DNA (Leal *et al.*, 2009 ▶); Se-Met containing FAE, feruloyl esterase module of xylanase 10B from *Clostridium thermocellum* (Prates *et al.*, 2001 ▶); RecR from *Deinococcus radiodurans* (Lee *et al.*, 2004 ▶); and the β_1_-adrenergic G-protein coupled receptor (GPCR) from *Meleagris gallopavo* (Warne *et al.*, 2008 ▶).

The measurements were carried out at the ESRF beamline ID23-1 (Nurizzo *et al.*, 2006 ▶), where the ADSC Q315 detector is installed. The beam size at the sample position is nominally 35 µm vertically and 45 µm horizontally (full width at half-maximum). At this beamline the incident-beam flux is continuously monitored and approximate calibration of the measurements to an absolute scale (photons s^−1^) is available over the full energy range. The beam energies and recorded values of the photon flux for the different crystals tested are given in Table 1 ▶. During these experiments the storage ring was operating in special filling modes, with maximum currents of 90 mA or 45 mA (for GPCR), and short life times. Changes in the operating modes may have affected both the beam size and monitor calibrations; no special measures were taken to correct for this effect.

To provide tests of the reproducibility of the procedure described in this work, for the large elongated crystals of thermolysin, trypsin, A-DNA and FAE, the procedure was carried out three to six times over the same sample, while translating unexposed parts of the crystal into the beam. Similarly, four very small crystals of RecR mounted in a single large nylon loop were probed. These crystals, obtained by Dr J. Radzimanowski under conditions previously used for crystallizing the *Deinococcus radiuodurans* RecO complex (Timmins *et al.*, 2007 ▶), belonged to a new body-centred orthorhombic crystal form. Only one of ten GPCR crystals, kindly provided by Dr M. Bowler, showed interpretable diffraction patterns. These could not be indexed in either a triclinic or a centred monoclinic lattice as previously published by Warne *et al.* (2008 ▶), but in a primitive monoclinic lattice as illustrate in Table 1 ▶. A knowledge of the exact symmetry is not essential for the method and was not determined for the GPCR crystal.

**Figure 1:**
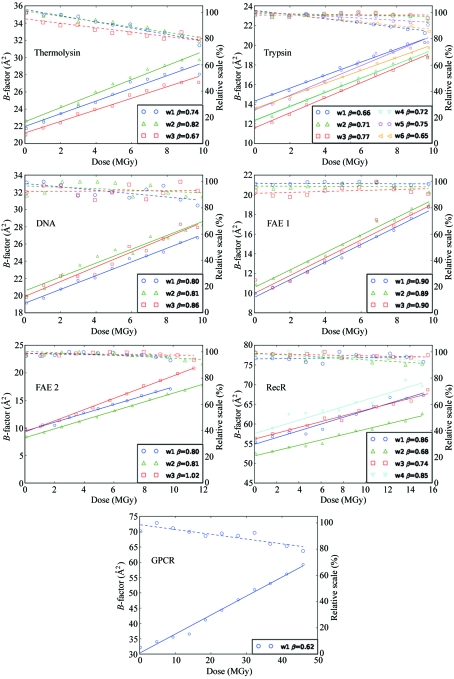
Relative scales (overall scale normalized to the maximum value for all the wedges) and *B*-factors against nominal dose. A linear fit of the *B*-factors *versus* dose is used to calculate the decay rate (or sensitivity coefficient) β. Full lines represent *B*-factors and dashed lines represent relative scales.

Bearing in mind the routine use of this method at the beamline, the procedure for dose calculations was applied without specifying the exact chemical composition of the sample, *i.e.* assuming the default composition for an average protein crystal (see Fig. 1 ▶). The resulting decay rate parameter β was then corrected according to the *RADDOSE* calculations using the known chemical composition of the sample (β_corrected_ in Table 2 ▶). The sample composition input, including the bound ions and solvent constituents, were defined according to the above literature references. For RecR and GPCR we assumed two and three protein molecules, respectively, in the asymmetric unit. An account was made for partially occupied Co sites present in A-DNA crystals (Leal *et al.*, 2010 ▶), whose occupancies summed up to four sites per asymmetric unit. For A-DNA and FAE the photon energies were chosen to be 10 eV below the absorption edges of Co and Se, respectively, thus avoiding any effects of near-edge features on the absorption cross sections.

The radiation damage data were integrated using *XDS*. In our hands, and for these particular conditions, short wedges of data collected under severe radiation damage, *XDS*, as compared with *MOSFLM*, produced superior sets of integrated intensities, as judged by the magnitude of random fluctuations in the estimated *B*-factors and scales (data not shown).

## 4. Discussion and conclusion

Overall, the results of the data analysis, as compiled in Table 2 ▶, and the observed variation of the scale and *B*-factor dependence on the dose presented on Fig. 1 ▶ for all experiments clearly confirm the practical applicability of the method described here. The linearity in the observed *B*-factor dependence on dose strongly supports the choice of the decay rate β as a generalized metric of damage. The relative changes in scale factors are significantly smaller and irreproducible. In none of the cases was the overall change of the scale factor at high resolution comparable with that of the *B*-factors. For all the automatically generated experiments the data collection protocol yielded experimental conditions appropriate to the correct sampling of this dependence, and ensured sufficiently accurate data sets down to a severe intensity decay level. This is observable in both the scatter of the *B*-factor values in Fig. 1 ▶ and in the 〈*J*〉/〈σ_*J*_〉 values given in Table 2 ▶, in particular for the very weakly diffracting RecR and GPCR crystals. Here, the rather high 〈*J*〉/〈σ_*J*_〉 and low resolution (for GPCR) are due to their very anisotropic diffraction: the orientation corresponding to the strongest diffraction was selected for data collection, whereas the choice of the resolution limit was based on the estimate of data set statistics over a full (spherical) resolution shell.

Furthermore, we note that, despite the very similar and systematic behaviour of intensity *versus* dose in all experiments, the variation in signal-to-noise, 〈*J*〉/〈σ_*J*_〉, as illustrated in Table 2 ▶, would not yield an effective metric of damage. This can be essentially attributed to the complexity in the relationships between signal and noise, as outlined by Bourenkov & Popov (2010 ▶). The studies of Kauffmann *et al.* (2006 ▶) and Nowak *et al.* (2009 ▶), both using the decay ‘*R*-factor’ space (*R*_d_) (Diederichs, 2006 ▶) as a metric of damage, reported significant variations in radiation sensitivity either as a function of radioprotecting scavengers or among individual crystals of identical preparations. This may also be attributed to the shortcomings of *R*_d_, which is essentially a signal-to-noise-based statistic, as a metric of radiation damage. Furthermore, the broad rotation ranges used in these experiments would unavoidably increase the variation in apparent damage rates.

The excellent reproducibility in the observed decay rates between the parts of the same crystal (thermolysin, trypsin, A-DNA, FAE) as well as between different crystals (FAE 1 *versus* FAE 2, RecR) suggests, with certainty, that in either of the two scenarios of a ‘sacrificial crystal’ experiment a single measurement suffices for accurate determination of the data collection strategy. Such an experiment would not be critically dependent on an accurate knowledge of sample composition (and hence the absorbance), nor on the precise beam parameter calibration. Although such an option appears very useful in everyday practice of data collection, we would like to stress that the method should not replace, or downscale, the importance of providing precise beam parameters to the users. The same refers to experimental characterization of sample chemical composition by various spectroscopic techniques, *e.g.* by microPIXE (Garman, 1999 ▶). These two components would be necessary prerequisites for interpretation of the decay rates on the ‘absolute’ dose scale, *i.e.* in terms of the radiation sensitivity of a particular system.

The selection of test systems presented here covers a broad range, not only in complexity, scattering power and crystal quality, but also in solvent and bound ion composition. The variation in β_corrected_ between different systems is again rather small, and comparable with the variation between the two ends and the central part of the long trypsin crystal. We attribute the observation of a β_corrected_ value systematically less than 1 Å^2^ MGy^−1^ to an inaccuracy in flux density calibration; most probably, the beam was larger than the nominal value by several micrometres. Owing to this factor, our data do not permit further generalizations.

The method itself has proven to be easy to use and requires minimal user interaction. In practice, the time required for decay characterization is defined by the photon flux density, *i.e.* a complete procedure could be accomplished in about a minute or two on a modern undulator beamline. The implementation is computationally efficient and will provide the final result a few seconds after the last frame was collected.

It is clear that the procedure has to be taken forward with a more accurate description of both the beam size and the beam shape, which may have strong effects on the observed intensity variations. We anticipate a new study where these issues will be fully addressed along with the complete automation of the method integrated into the *EDNA* platform.

## Figures and Tables

**Table 1:** Crystal characteristics and experimental set-up for the samples used in this work

Crystal	Unit cell (Å)	Space group	Crystal size (µm)	Energy (keV)	Flux (photons s^−1^)	Dose rate (MGy s^−1^)	Total dose (MGy)	Resolution limit (Å)
Thermolysin	*a* = *b* = 93.16, *c* = 129.31	*P*6_1_22	300 × 50 × 50	12.76	6.2 × 10^11^	0.15	∼10	2.0
Trypsin	*a* = 61.87, *b* = 63.66, *c* = 68.68	*P*2_1_2_1_2_1_	900 × 100 × 100	12.76	9.9 × 10^11^	0.24	∼10	2.0
A-DNA	*a* = *b* = 32.65, *c* = 77.76	*P*6_1_22	400 × 200 × 200	7.70	2 × 10^11^	0.14	∼10	2.0
FAE 1	*a* = 65.4, *b* = 108.5, *c* = 113.6	*P*2_1_2_1_2_1_	400 × 40 × 40	12.64	8.0 × 10^11^	0.20	∼10	2.0
FAE 2	*a* = 65.4, *b* = 108.5, *c* = 113.6	*P*2_1_2_1_2_1_	400 × 40 × 40	12.64	1.0 × 10^12^	0.24	∼10 to 12	2.0
RecR	*a* = 71.52, *b* = 71.95, *c* = 174.05	*I*222	20 × 20 × 20	12.75	7.6 × 10^11^	0.17	∼15	3.0
GPCR	*a* = 89.18, *b* = 60.98, *c* = 101.01	*P*2, β = 109.4°	50 × 40 × 10	12.72	4.3 × 10^11^	0.10	∼46	4.2

**Table 2:** Crystal sensitivity statistics determined following the radiation damage protocol described in this work The values are organized by sample and the position (Pos) at which the data were collected. The columns 〈*J*〉/〈σ_*J*_〉_i_ and 〈*J*〉/〈σ_*J*_〉_f_ represent the signal-to-noise ratio in the last resolution shell for the first and last wedges. The last column shows the β value corrected according to *RADDOSE* calculations using the known chemical composition of the samples. The β values are given in Å^2^ MGy^−1^. The standard deviation was calculated according to the formula 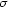 = 
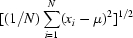
, where μ is the average value.

Crystal	Pos	Mosaicity (°)	〈*J*〉/〈σ_*J*_〉_i_	〈*J*〉/〈σ_*J*_〉_f_	β	β_average_	β_std.deviation_	β_corrected_
Thermolysin	1	0.56	5.2	2.0	0.74	0.74	0.06	0.57
	2	0.51	3.9	1.6	0.82			0.63
	3	0.53	4.6	2.3	0.67			0.52
Trypsin	1	0.54	12.1	5.1	0.66	0.71	0.04	0.61
	2	0.61	13.4	5.2	0.71			0.66
	3	0.69	15.2	5.5	0.77			0.71
	4	0.63	16.8	6.3	0.72			0.67
	5	0.53	13.4	5.6	0.75			0.69
	6	0.63	15.8	7.3	0.65			0.60
A-DNA	1	0.32	13.3	8.1	0.80	0.82	0.03	0.61
	2	0.40	8.6	6.6	0.81			0.62
	3	0.46	8.8	7.6	0.86			0.66
FAE 1	1	0.25	9.1	3.6	0.90	0.9	0.01	0.63
	2	0.23	9.0	3.6	0.89			0.62
	3	0.17	6.5	3.4	0.90			0.63
FAE 2	1	0.31	9.3	3.9	0.80	0.88	0.1	0.56
	2	0.27	12.5	4.5	0.81			0.57
	3	0.25	7.4	2.1	1.02			0.71
RecR	1	0.88	4.3	1.9	0.86	0.78	0.08	0.76
	2	0.90	5.1	2.7	0.68			0.60
	3	0.60	4.5	1.9	0.74			0.65
	4	0.86	3.9	1.9	0.85			0.75
GPCR	1	0.80	15.1	6.8	0.62	0.62	NA	0.64
